# A comprehensive review on the hepatotoxicity of herbs used in the Indian (Ayush) systems of alternative medicine

**DOI:** 10.1097/MD.0000000000037903

**Published:** 2024-04-19

**Authors:** Cyriac Abby Philips, Arif Hussain Theruvath

**Affiliations:** a Clinical and Translational Hepatology, The Liver Institute, Center of Excellence in Gastrointestinal Sciences, Rajagiri Hospital, Aluva, India; b Department of Clinical Research, Division of Complementary and Alternative Medicine and the Liver, The Liver Institute, Center of Excellence in Gastrointestinal Sciences, Rajagiri Hospital, Aluva, India.

**Keywords:** acute liver failure, acute on chronic liver failure, Ayurveda, hepatotoxicity, herb-induced liver injury, herbal

## Abstract

Complementary and alternative medicine-related liver injuries are increasing globally. Alternative medicine, as an inclusive healthcare practice, is widely accepted in developing and underdeveloped countries. In this context, the traditional systems of medicine in India have been at the forefront, catering to the preventive and therapeutic spectrum in the absence of conclusive evidence for benefits and lack of data on safety. Contrary to popular belief, it is evident that apart from adverse events caused by contamination and adulteration of alternative medicines, certain commonly used herbal components have inherent hepatotoxicity. This narrative review updates our current understanding and increasing publications on the liver toxicity potential of commonly used herbs in traditional Indian systems of medicine (Ayush), such as *Tinospora cordifolia* (Willd.) Hook.f. & Thomson (Giloy/Guduchi), *Withania somnifera* (L.) Dunal (Ashwagandha), *Curcuma longa* L. (Turmeric), and *Psoralea corylifolia* L. (Bakuchi/Babchi). This review also highlights the importance of the upcoming liver toxicity profiles associated with other traditional herbs used as dietary supplements, such as *Centella asiatica* (L.) Urb., *Garcinia cambogia* Desr., *Cassia angustifolia* Vahl (Indian senna), and *Morinda citrofolia* L. (Noni fruit). Fortunately, most reported liver injuries due to these herbs are self-limiting, but can lead to progressive liver dysfunction, leading to acute liver failure or acute chronic liver failure with a high mortality rate. This review also aims to provide adequate knowledge regarding herbalism in traditional practices, pertinent for medical doctors to diagnose, treat, and prevent avoidable liver disease burdens within communities, and improve public health and education.

## 1. Introduction

Indian systems of traditional and alternative medicine are currently grouped under the acronym Ayush, which stands for Ayurveda, Yoga and Naturopathy, Unani, Siddha, and Homeopathy. Ayurveda is an ancient, prescientific observation-based compendium of healthcare practices originating nearly 2000 years ago, which, apart from a multitude of “holistic” practices, also involves the prescription of crude herbs, herbal mixtures, and liquor, and herbo-mineral and metallic preparations to treat a wide range of symptoms.^[1,2]^ Similar use is noticeable in Siddha, a traditional medical practice akin to Ayurveda, originating in South India, catering to the population in the state of Tamil Nadu.^[3]^ Unani medicine is a Persian/Arabic traditional healthcare practice with origins in the doctrines of ancient Greek physicians, currently exclusive to Islamic culture in Central and South Asia.^[4]^ Even though Homeopathy is included within the traditional medicine spectrum of Ayush, it is neither traditional nor Indian in origin. Homeopathy is an unscientific system of medical practice based on fallacious principles, with origins in Germany in the early 1800s. It proposes the benefits of using ultra-diluted formulations or alcohol-based mother tinctures to treat a wide range of symptoms and prevent various diseases in the absence of conclusive evidence for benefits.^[5,6]^ The use of crude herbs and multiherbal formulations is common to these alternative medical systems. A growing body of published evidence demonstrates that liver injury and liver failure from traditional and complementary and alternative medicine (CAM) practices, necessitating liver transplantation, has been on the rise.^[7,8]^ The prevalence of overall CAM use was 24%, 50%, and 71.3% in Switzerland, Germany, and South Korea, respectively. The prevalence of CAM use among the cirrhotic population in India was 63%. The mortality rates due to CAM liver injury are estimated to be between 5% and 10% (in Chinese, Singapore, and Japanese series) to 10% to 19% (in Indian studies) in those without underlying liver disease, and >50% in those with preexisting liver disease or severe liver disease. Ayurvedic herbal formulations can cause liver injury that adversely affects the natural course of severe liver disease and worsen preexisting liver disease, leading to acute-on-chronic liver failure (ACLF) with high fatality.^[7–9]^ Furthermore, a large series of patients with “Ayush-immune boosting” and homeopathic remedies-related liver injury were recently published from the Indian subcontinent.^[10,11]^ The Asia-Pacific Association for Study of Liver (APASL) drug-induced liver injury (DILI) guidelines faculty coined the term “Ayush-liver injury” for liver injury originating specifically from Indian CAM.^[12]^ A multicentre study from the APASL demonstrated that ACLF in Asia-Pacific countries was predominantly due to CAM (inclusive of Ayurvedic herbs and herbal and dietary supplements) in approximately 72% of patients.^[13]^ Recent studies have shown an 8-fold increase in liver transplantation due to herbal and dietary supplements in the West, and an increased requirement for liver transplantation for acute liver injury from herbal supplements among Asian communities in the West.^[14,15]^ In this narrative review, we exhaustively discuss the potential hepatotoxicity of specific herbs utilized in Ayush practice from the peer-reviewed published medical literature.

## 2. Methods

An extensive search was conducted in PubMed, Web of Science, and Scopus databases for articles published between 2013 and 2023. Keywords used included “Ayush,” “Ayurvedic herbs,” “Homeopathy liver injury,” “Ayush liver injury,” “herbal liver injury,” “herb induced liver injury,” “HILI,” “DILI due to herbs,” “herbal hepatotoxicity,” “herbal liver toxicity,” “herbal liver failure,” “herb induced liver failure,” “acute liver failure due to herbs,” “liver failure due to herbs,” “herbal supplements and liver failure,” and “liver toxic herbs.” The search included case reports and series, clinical trials, observational studies, and systematic and narrative reviews specifically related to and in the context of the Indian subcontinent, with no restrictions on publications, journals, or languages. Utilizing the SANRA Narrative Review Article Quality Assessment Scale, 2 authors screened abstracts and full texts to identify relevant research on herb-induced liver injury (HILI) within the Indian context. Disagreements were resolved via discussion. All selected articles were thoroughly reviewed, and references were examined. After a thorough literature search and screening, publications from 2013 to 2023 were finally used in this review, and the data were organized to focus and narratively summarize findings under specific herbs and miscellaneous herbs related to liver toxicity. Additionally, a discussion on liver failure outcomes due to herbs and herbal supplements was also provided. Given the nature of this literature review, institutional ethical approval was not necessary.

## 3. Specific herbs and their toxicity

### 3.1. *Tinospora cordifolia* (Willd.) Hook.f. & Thomson *(Giloy, Guduchi*)

**Figure 1.**
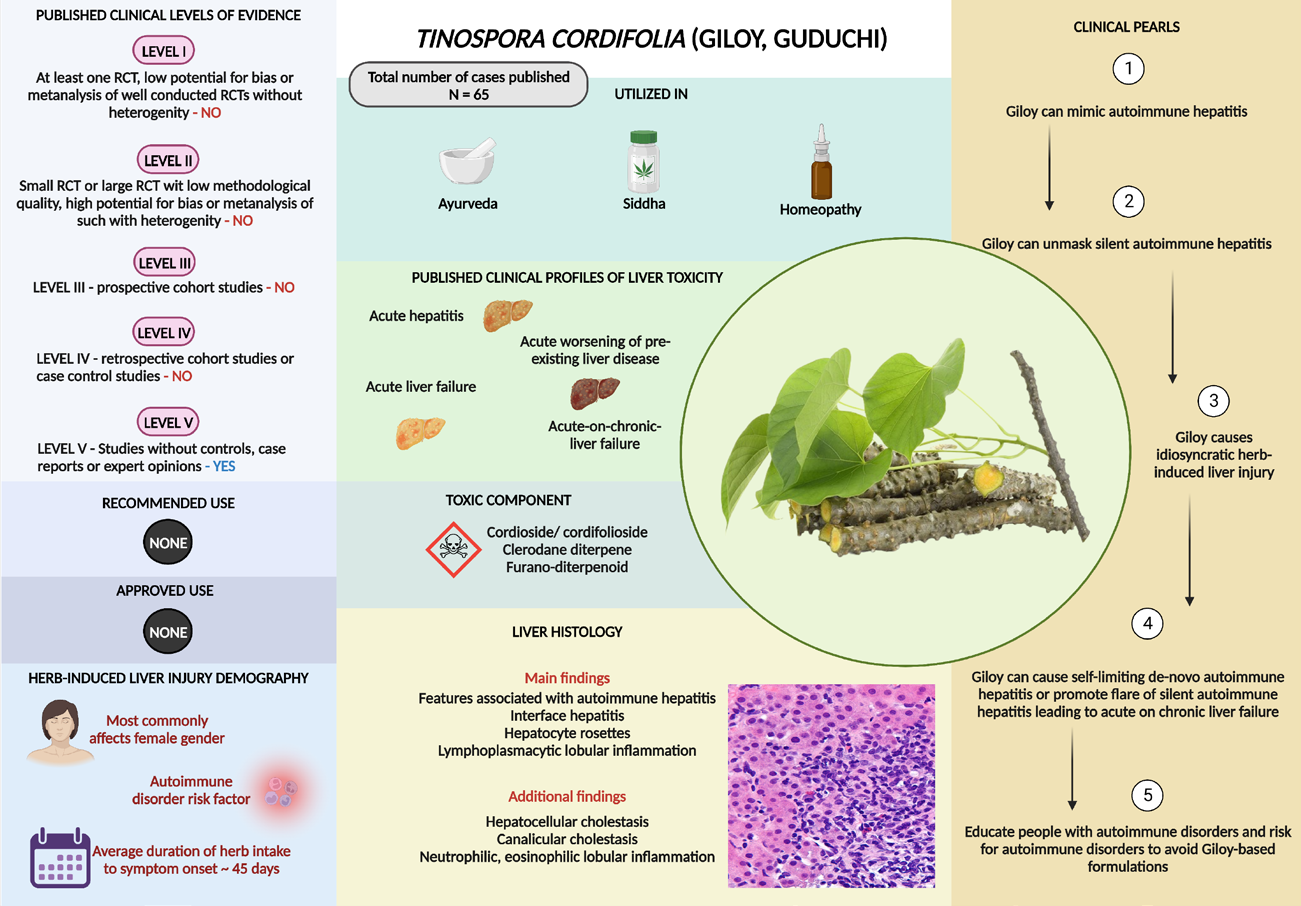
Summary of *Tinospora cordifolia* (Willd.) Hook.f. & Thomson (Giloy) related liver injury.

*Tinospora cordifolia* (Willd.) Hook.f. & Thomson known as Giloy (in Hindi) or Guduchi (in Sanskrit), is commonly called heart-leaved moonseed. This herb has been used for millennia as part of various traditional and proprietary herbal formulations in Ayurvedic and Siddha practice. The roots, stems, and leaves of Giloy are used in folk medicine to treat several disorders, in the absence of significant therapeutic benefits.^[16]^ The plant contains highly bioactive phytocompounds such as alkaloids (berberine, palmatine, and tinosporin), diterpenoid lactones (furanolactone and clerodane derivatives), glycosides (furanoid diterpene glucoside and tinocordiside), steroids (sitosterol and giloinsterol), and aliphatic (octacosanol and nonacosan-dichloromethane) chemicals. Although these compounds have been shown to have anti-inflammatory, antioxidant, anti-hypoglycemic, antispasmodic, antineoplastic, and immunomodulatory effects in preclinical studies in cells, tissues, and small animals, clinically relevant benefits in humans have not been confirmed through rigorous trials. The current use of Giloy in CAM practice is shouldered on belief and faith by appealing to tradition and antiquity and not based on scientific evidence.^[17]^ Giloy is currently the highest reported causative agent of HILI in India. Hepatologists at the Jaslok Hospital and Research Centre, Mumbai, were the first to report 6 patients with Giloy herb consumption and acute hepatitis.^[18]^ Giloy-induced autoimmune-like hepatitis or unmasked underlying silent autoimmune chronic liver disease due to its immune stimulant mechanism resulting in severe liver injury. Proponents of Ayurveda were quick to dismiss these cases as adulteration with another similar hepatotoxic herb, *Tinospora crispa* (L.) Hook.f. & Thomson (TC) also documented to cause fulminant hepatitis.^[19,20]^ However, the authors collected 4 plant samples and 2 commercial preparations that were consumed by their patients and subjected them to high-performance thin-layer chromatography phytochemical analysis and DNA barcoding studies, which confirmed the causative herb Giloy and not TC.^[21]^ The largest series of Giloy-induced HILI, a nationwide multicenter study was reported by the Liver Research Club India (LIVERECI).^[22]^ Of 43 patients, >50% were females, presenting with acute hepatitis, acute worsening of chronic liver disease, or acute liver failure. The chemical and toxicological analyses of the retrieved Giloy samples did not reveal any contamination or adulteration. The study concluded that Giloy was associated with autoimmune-like hepatitis and that the herb had the potential to unmask autoimmune hepatitis (AIH) in people with silent AIH-related preexisting liver disease. Reports of Giloy-related HILI have been consistently reported in many parts of the world, but mostly from the Indian subcontinent (Table 1, Supplemental Digital Content, http://links.lww.com/MD/M220).^[23–28]^ Liver biopsy usually reveals features associated with autoimmune hepatitis (interface hepatitis, hepatocyte rosettes, and lymphoplasmacytic lobular inflammation), hepatocellular and canalicular cholestasis, and additional portal or lobular neutrophilic and eosinophilic infiltration.^[22]^ Both Giloy and TC contain cis-clerodane-type furano-diterpenoids that have the potential to induce hepatotoxicity, and the immune-stimulating properties of Giloy have been well documented. This immune stimulation is possibly due to the presence of plant-based alkaloids, glycosides, and terpenoids, such as cordifoliosides, that promote increased polyclonal immunoglobulin G production from B-cells, demonstrably associated with AIH.^[29]^ Death due to Giloy-induced HILI is rare, and patients respond well to conservative care and a short course of steroids. Nonetheless, fatality due to acute worsening of underlying cirrhosis and the need for liver transplantation in patients with acute liver failure were reported.^[22]^ To summarize, in the absence of substantiated clinically relevant benefits, Giloy can promote the development of life-threatening hepatitis, especially in those with diagnosed or silent autoimmune liver disorder or who are at risk of developing autoimmune diseases (Fig. 1).

### 3.2. *Withania somnifera* (L.) Dunal *(Ashwagandha*)

*Withania somnifera* (L.) Dunal popularly known as Ashwagandha (Indian Winter Cherry or Indian Ginseng) is an important and commonly used herb in traditional Ayurvedic supplements. Considered a health-promoting tonic as per classical Ayurvedic texts it is prescribed for a wide range of health and disease conditions.^[29]^ Even though Ashwagandha is claimed to have health benefits and therapeutic effects (increasing longevity in the elderly, improving immune status, antianxiety, improving recovery from fatigue, increasing memory, and reproductive function), there is a lack of validated clinical evidence from well-designed and rigorously performed studies that conclusively prove its safety or efficacy.^[30]^

**Figure 2.**
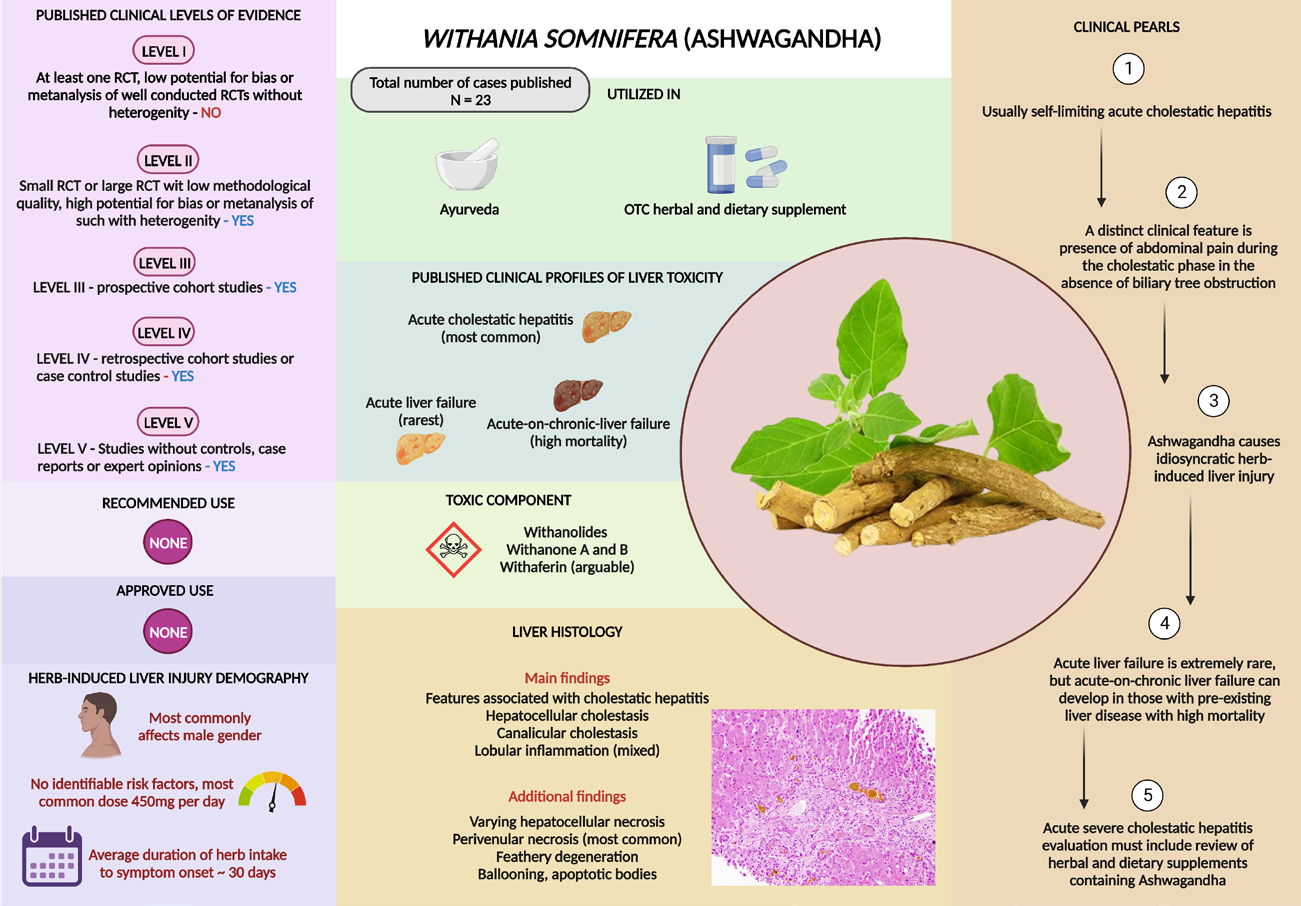
Summary of *Withania somnifera* (L.) Dunal (Ashwagandha) related liver injury.

Flavonoids, phytosteroids, coagulins, alkaloids such as cuscohygrine and anahygrine, and steroidal lactone triterpenoids, also known as withanolides, are among the bioactive substances found in Ashwagandha. There are unsubstantiated claims that the terpenoid withaferin-A has hepatoprotective qualities. The first report of Ashwagandha-induced HILI was from Japan in a 20-year-old male with anxiety disorder.^[31]^ Severe intrahepatic cholestasis with widespread canalicular bile plugs was notable on liver biopsy. After receiving treatment with ursodeoxycholic acid and phenobarbitone and stopping the offending medication, the patient recovered uneventfully in less than 2 months. Björnsson et al^[32]^ reported several patients with Ashwagandha-induced liver damage from Iceland and the US Drug-Induced Liver Injury Network (DILIN). The authors reported 5 patients, with a mean age of 43 years, who experienced cholestatic jaundice 2 to 12 weeks after taking Ashwagandha supplements. A liver biopsy revealed significant cholestatic hepatitis. The length of the clinical course was 5 to 20 weeks, and it took on average 5 months for the clinical symptoms and signs to abate. None of the patients developed liver failure. Chemical examination of the recovered formulations using liquid chromatography coupled with quadrupole time-of-flight mass spectrometry verified the presence of Ashwagandha and the absence of other harmful substances or liver-toxic prescription drugs. The LIVERECI group recently published the largest series of Ashwagandha-related HILI.^[33]^ The authors described 8 individuals with HILI truly attributable to a single-component formulation among 23 patients who experienced liver damage from consuming Ashwagandha-based supplements. Men predominated, and the commonest presentation was cholestatic hepatitis. Three patients who presented with ACLF died during the follow-up. Others experienced prolonged but self-limiting liver damage. Liver biopsy revealed cholestatic features, including lymphocytic and eosinophilic portal-based inflammation, and varying types of hepatocellular necrosis in the majority. One patient developed chronic HILI with waxing and waning liver test abnormalities with evolution to chronic liver disease during follow-up. Only natural phytochemicals were detected upon chemical examination of the retrieved Ashwagandha formulations, which were essentially free of contamination or adulteration. Although rare, Ashwagandha-related HILI is currently being reported frequently worldwide due to increased promotion and exaggerated advertisements, based on inconclusive evidence (Table 2, Supplemental Digital Content, http://links.lww.com/MD/M221). The Netherlands Pharmacovigilance Center Lareb reported 4 cases while the World Health Organization pharmacovigilance database [Vigibase] documented 15 HILI reports related to Ashwagandha.^[34]^ The cause of hepatotoxicity due to Ashwagandha is arguably due to irreversible adduction of withanolide to hepatocellular DNA, subsequent cellular damage, excessive excitation of hepatocytes, and overstimulated depletion of glutathione levels, which translates to liver damage.^[35,36]^ In 2020, the Technical University of Denmark notified that it was not possible to establish a standardized safe lower limit for Ashwagandha in supplements. Denmark also banned Ashwagandha because of its potential negative effects on hormones and ability to induce abortion apart from the hepatotoxicity. Subsequently, German and Swedish health regulatory agencies reportedly concurred with the Danish risk assessment, generating the possibility that other European Union member states may ban Ashwagandha-based supplements in the near future.^[37,38]^ In summary (Fig. 2), once considered rare, reports Ashwagandha-related HILI are on the rise globally. Patients present with prolonged cholestatic hepatitis with an increased risk of death notably in those with cirrhosis.

### 3.3. *Curcuma longa* L. *(Turmeric*)

**Figure 3.**
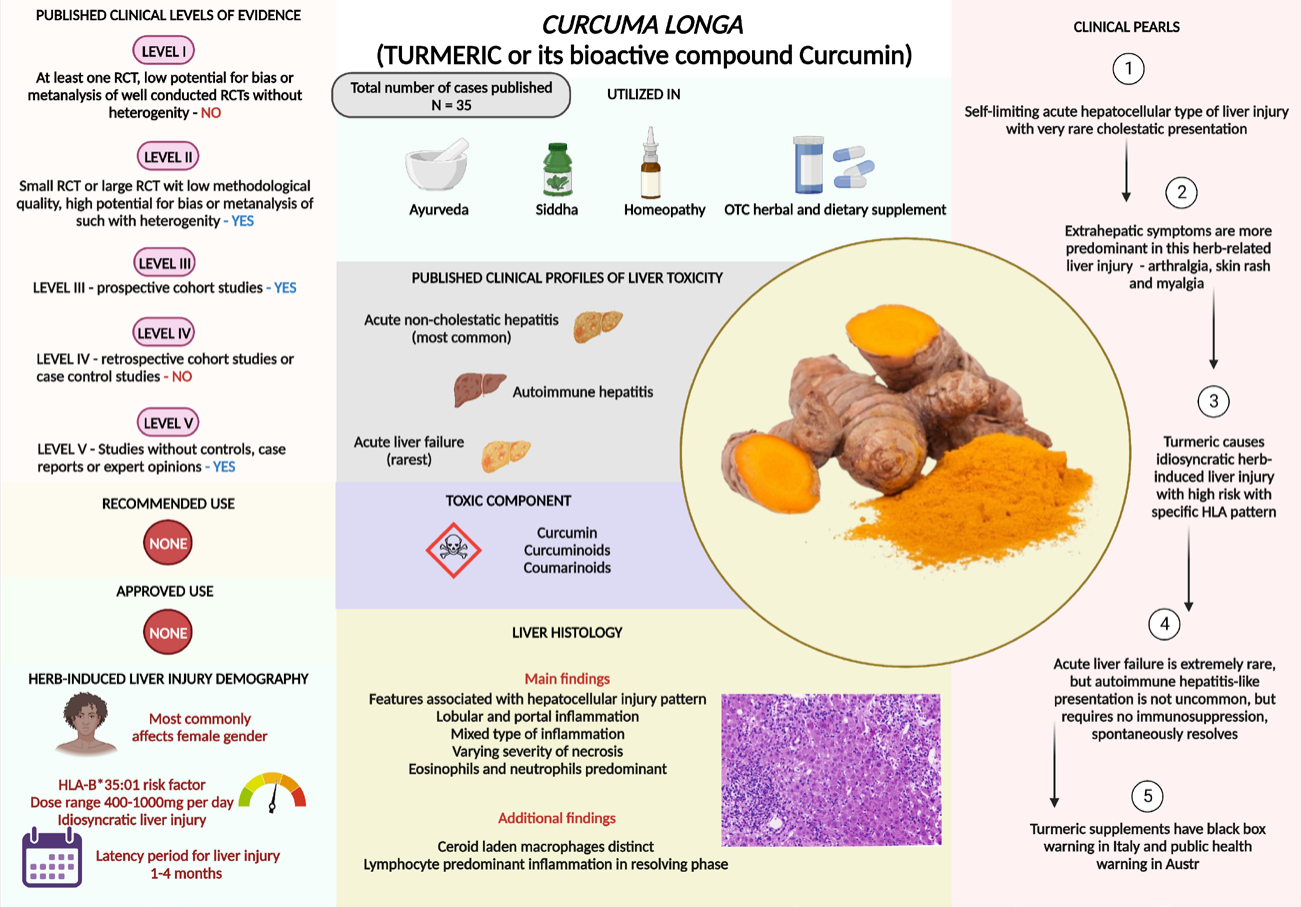
Summary of *Curcuma longa* L. (Turmeric/curcumin) related liver injury.

*Curcuma longa* L. belongs to the Zingiberaceae (ginger) family. The commonly used part of this plant is the rhizome (the main stem of the plant that runs underground horizontally), which contains a wide variety of bioactive compounds. These include nonvolatile curcuminoids, such as bisdemethoxy- and dimethoxy curcumin and curcumin, along with volatile oils, such as mono- and sesquiterpenoids.^[39]^ Turmeric or its bioactive compound curcumin in highly bioavailable forms, is used in traditional Ayurvedic preparations or as dietary supplements, respectively.^[40]^ The bioavailability of turmeric is <1%; that is, when turmeric is directly consumed, 99% of it is excreted in stools, resulting in negligible circulating amounts for effective systemic actions. Furthermore, the half-life of turmeric (considering its most bioactive compound, curcumin) is very short, without bioavailability after 5.45 hours. Turmeric contains up to 5% curcumin; which means, it contains only 5% of its bioactive component, and only 1% of that 5% is available for action in the body when turmeric is consumed orally. This is extremely small for turmeric to exhibit significant biological activity in humans.^[41]^ Thus, the small quantity of turmeric that traditionally people put into their hot water or milk or consume on empty stomach for supposed health benefits is devoid of any biological effects. Curcumin is a pan-assay interference compound or PAIN. The PAIN compounds show different types of activity in assay experiments, which are wrongly attributed to the “significant” activity of the compound tested. These bioactivities are not due to interactions of the study compound (e.g., curcumin) with cells or tissues, but are deceptive actions that occur with other chemical components in the experiment assay.^[42]^ This is one reason why major research groups studying turmeric or curcumin have failed to consistently replicate clinical experiments, and meta-analyses have always pointed towards the need for better studies on turmeric outside of preclinical trials. Turmeric and curcumin are also “invalid metabolic panaceas (IMPs).” IMP compounds show false promise in early experiments, and have negligible or no effects when studied inside a controlled environment, such as in a clinical experiment or trial.^[43]^ Turmeric and its bioactive components are also meritless “lead compounds.” Lead compounds are those that have potential for therapeutic discovery and transformation into drugs for clinical use. Ideal lead compounds have good potency at low concentrations, are chemically stable with high bioavailability, and are identifiable with selective mechanisms of action all of which are lacking in turmeric.^[44]^ To improve bioavailability, concentrated forms of turmeric and curcumin, including combinations with black pepper/piperine, lipid complexes, nano-emulsions, and liposomal mixtures, are currently available in the market. These formulations have led to an increase in the number of published reports on turmeric-based liver injuries. Initially, these reports were isolated and occurred in various parts of the world and have been consistently reported (Table 3, Supplemental Digital Content, http://links.lww.com/MD/M222). Two major studies have clarified realistic liver injuries due to turmeric supplements that have resulted in regulatory actions from various healthcare authorities. An analysis of the Italian Phytovigilance database and a systematic review of case reports revealed 7 cases of acute hepatitis occurring in Tuscany, where hepatotoxicity was associated with highly bioavailable turmeric formulations.^[45]^ The causal relationship was also supported by the positive de-challenge observed in most cases, and 23 cases identified through the systematic review demonstrated that patients were concomitantly exposed to at least one other medication, and 16 of them experienced a positive de-challenge. This led to the Italian government prohibiting all health claims linked to turmeric and issuing warnings for labels on turmeric-based dietary supplements.^[46]^ Similarly, the United States DILI Network reported 10 cases of turmeric-associated liver injury among 8 women with a median age of 56 years and the growing problem of liver injury associated with turmeric formulations.^[47]^ Liver injury was predominantly of the hepatocellular type, and histopathology revealed acute hepatitis or mixed cholestatic hepatic injury with eosinophils. Five patients were hospitalized, and one died of acute liver failure. Chemical analysis confirmed the presence of turmeric in all 7 products tested, and 3 products also contained piperine (black pepper). HLA typing demonstrated that 7 patients carried HLA-B*35:01, which was considered a risk factor for idiosyncratic liver injury from turmeric supplements and was also notable for liver injury related to green tea extracts.^[48]^ Recently, the Australian Government, Department of Health and Aged Care, Therapeutic Goods Administration (TGA) responsible for evaluating, assessing, and monitoring therapeutic products, as well as regulating medicines, medical devices, and biologicals, warned the public about the risk of liver injury from turmeric-based dietary supplements. The TGA also stated that the risk was also related to other ingredients from the *Curcuma* species (since they contain bioactive curcumin), such as *Curcuma aromatica, Curcuma zanthorrhiza*, and *Curcuma zedoaria*.^[49]^ In summary (Fig. 3), even though rare, the rise in liver injury from turmeric supplements is on the rise globally due to extensive promotion of its clinical benefits exaggeratingly extrapolated from preclinical studies. Persons with specific genetic risk factors are predisposed, and liver injury is usually hepatocellular in type and self-limiting, but with the potential to progress to fatal acute liver failure.

### 3.4. *Psoralea corylifolia* L. *(Bakuchi or Babchi*)

**Figure 4.**
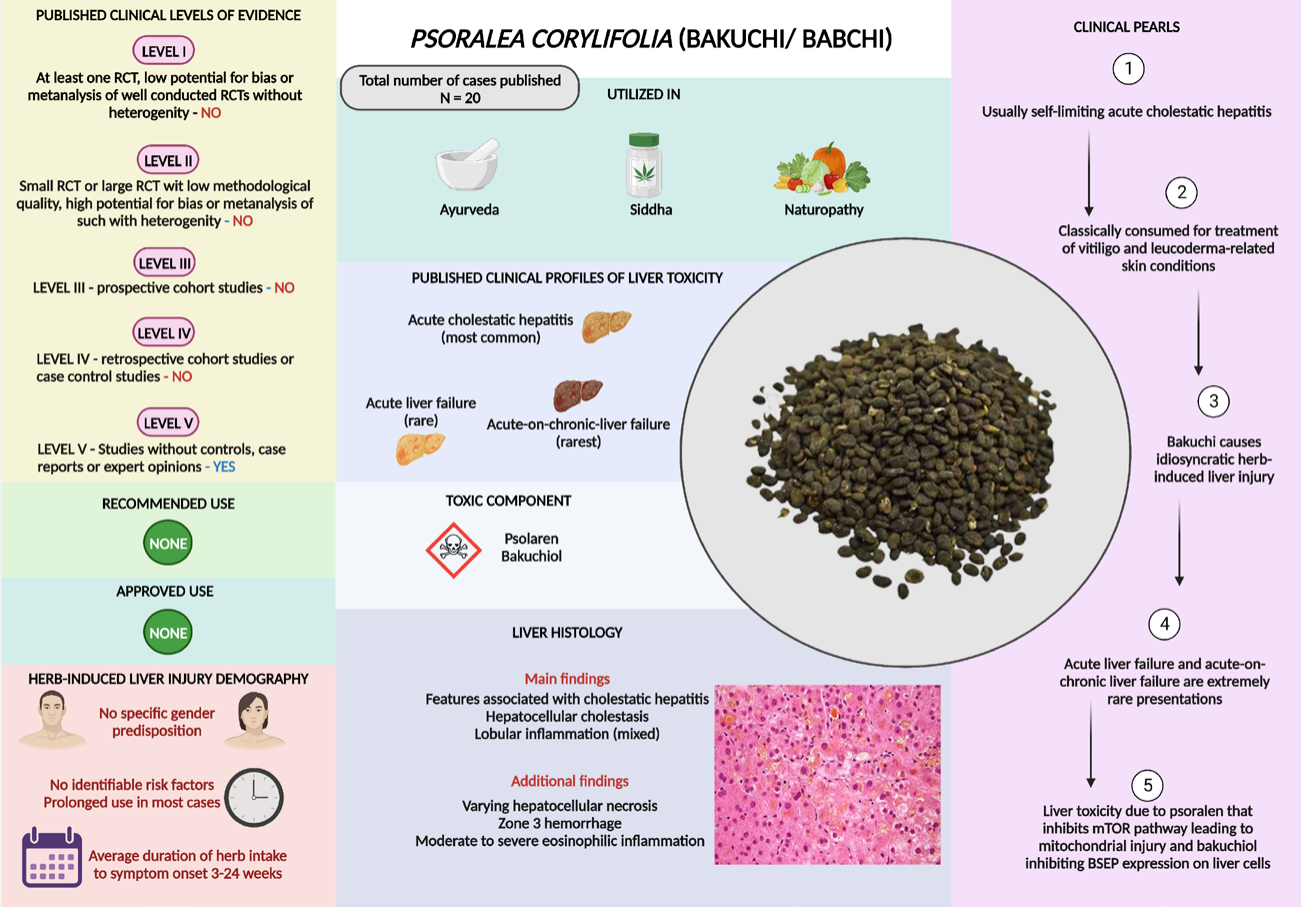
Summary of *Psoralea corylifolia* L. (Bakuci/babchi) related liver injury.

*Psoralea corylifolia* L. (purple fleabane) a species of the Leguminosae (or Fabaceae) family, also known as Bakuchi or Babchi, is commonly used as an alternative medicine in Ayurvedic medicine for the treatment of skin disorders, such as psoriasis, leukoderma, and leprosy, mostly in the form of oral formulations.^[50]^ The commonly used are seeds (called *Fructus psoraleae* L.), which contain the most bioactive compounds, including flavones, coumarins, monoterpenes, chalcones, lipids, resins, stigmasteroids, and flavonoids. Bakuchiol is a monoterpene component that is thought to have therapeutic effects. Other bioactive compounds of interest include astragalin, corylifols (flavonoids), bavachinin (flavone), bakuchinin (coumarin), and psolaren (furanocoumarin). Like most other herbs of interest in Ayurvedic literature, Bakuchi has also shown promising anti-inflammatory, antioxidant, antitumor, antimicrobial, and immunomodulating properties in the preclinical setting without any clinically relevant benefits in human trials.^[51]^ In the absence of clinical evidence, Bakuchi has been widely used in the alternative medicine industry, mostly for the treatment of depigmentation-related skin conditions such as vitiligo and leukoderma. The psoralens in Bakuchi have been shown to inhibit the mammalian target of rapamycin (mTOR) signaling pathway, cause mitochondrial injury, and impaired liver regeneration, with negative effects on lipid metabolism, leading to its well-documented hepatotoxicity. Furthermore, dose-related and frequency-related accumulation of psoralen has demonstrated hepatotoxicity in small animals and in vitro studies.^[52]^ Similarly, the most highly bioactive component, bakuchiol, was found to cause cholestatic hepatitis in treated rats by downregulating the expression of the bile salt export pump (BSEP) protein in liver cells. Bakuchiol-treated rats showed suppression of weight gain and food intake, alteration in liver tests, swelling, and increased liver weight.^[53]^ There are a total of 8 cases of Bakuchi-related liver injury reported to date in the medical literature which also included 1 patient who developed ACLF after prolonged use of the seed-formulation for vitiligo.^[54]^ Acute liver failure and death due to Bakuchi use have also been reported (Table 4, Supplemental Digital Content, http://links.lww.com/MD/M223). Liver injury due to Bakuchi is usually self-limiting, and the overall prognosis of Bakuchi liver injury is good. Nonetheless, although rare, fatality in those with comorbidities, including preexisting liver disease, has been documented. To summarize, Bakuchi-related liver injury presents predominantly as cholestatic hepatitis in those with prolonged use of seed-based formulations for chronic dermatological conditions, such as vitiligo, and may cause progressive liver failure, leading to a high risk of death in patients with underlying liver disease or other chronic comorbid conditions (Fig. 4).

## 4. Miscellaneous herbs and their toxicity

**Figure 5.**
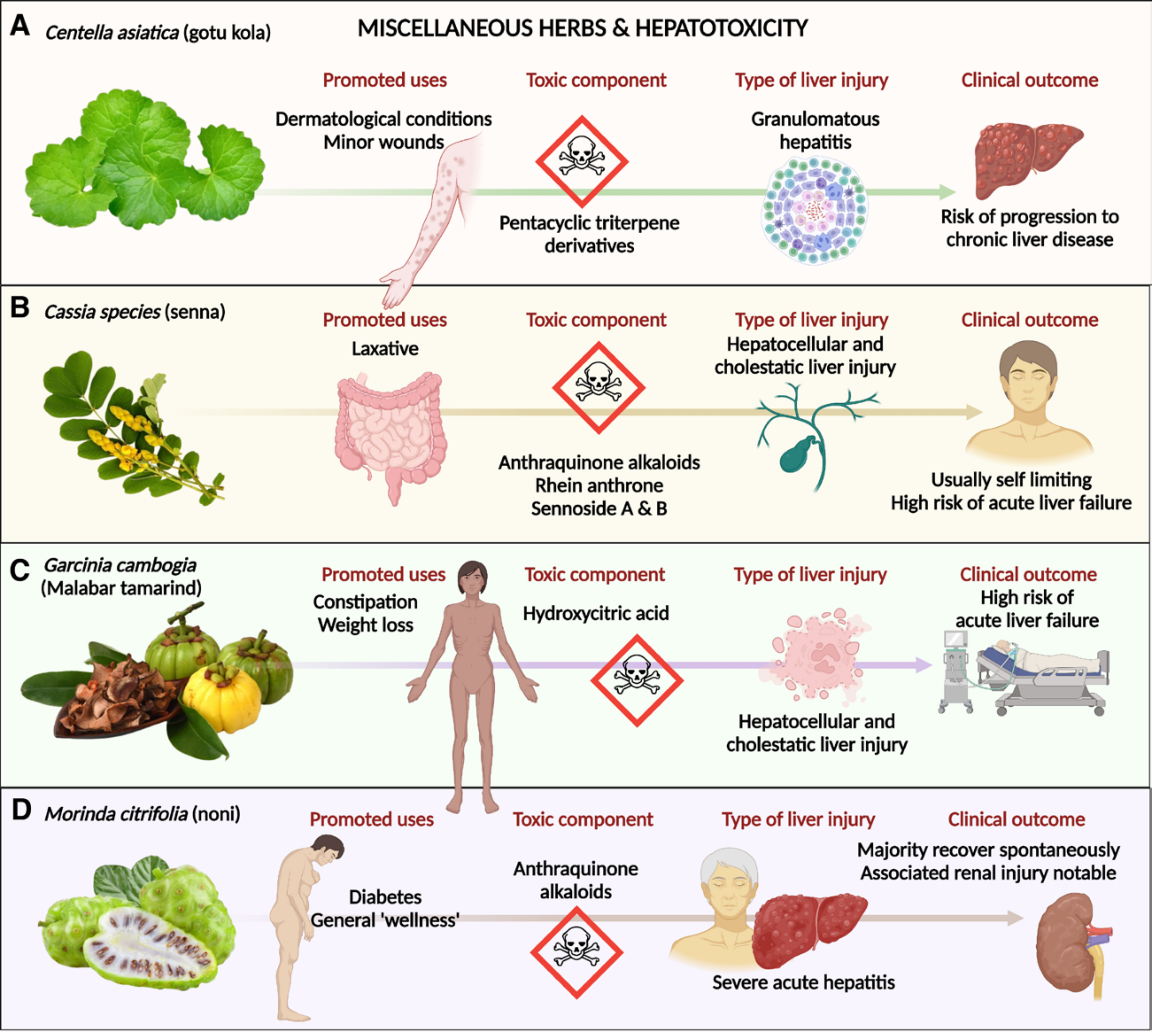
Summary of hepatotoxicity of other herbs. (A) *Centella asiatica* (L.) Urb. (Gotu kola), (B) *Cassia angustifolia* Vahl (Indian senna), (C) *Garcinia cambogia* Desr. (Malabar tamarind), and (D) *Morinda citrofolia* L. (Noni fruit).

**Figure 6.**
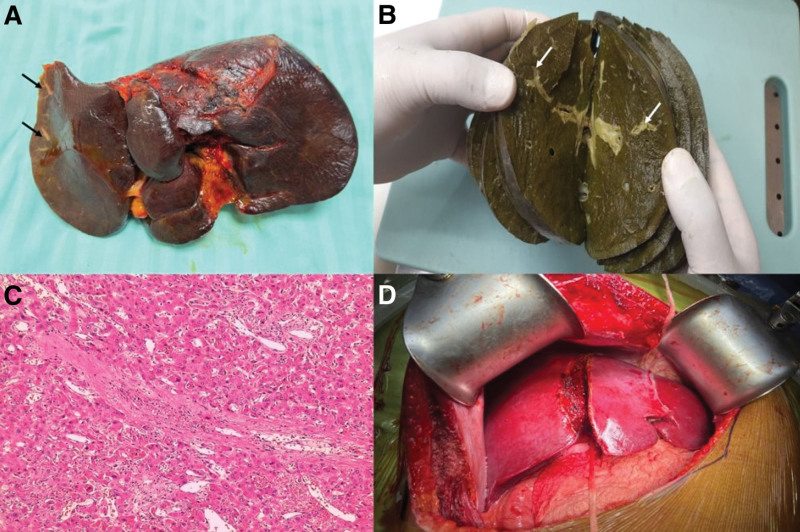
Hepatic sinusoidal obstruction syndrome due to ingestion of Ayurvedic herbs containing pyrrolizidine alkaloids. A 56-year-old man was prescribed herbal formulations for anxiety by an Ayurvedic healer. Two weeks later, he developed abdominal pain, swelling of legs, ascites, pleural effusion and worsening hepatocellular jaundice. Conservative treatment did not improve his condition, culminating in liver transplantation. Explant liver was enlarged, bluish, with capsular stretching, and visibly sclerosed hepatic venules (A, B, arrows). Biopsy revealed central vein occlusion (C), sinusoidal hemorrhage and hepatic necrosis, suggestive of veno-occlusive disease. The healthy living-donor liver is shown for comparison (D).

1. *Centella asiatica* (L.) Urb., or the Indian pennywort (gotu kola in Sri Lanka, jalbrahmi, or mandukparni in Ayurvedic practice), belonging to the flowering plant family Apiaceae, has been used in traditional medicine for various dermatological disorders, such as leprosy, psoriasis, varicose veins, varicose skin ulcers, and treatment of minor wounds. Nonetheless, its clinical efficacy and safety remain to be proven under these conditions. Although gotu kola has been shown to protect against liver damage from oxidant injury in preclinical studies, appropriate and adequate clinical studies that unmask its benefits remain unknown. In a systematic review of the profile of herbal and dietary supplements induced liver injury in Latin America, *Centella asiatica* (L.) Urb. was the most reported culprit, along with Herbalife® products and *Carthamus tinctorius* L.^[55]^ The most common (unsubstantiated) indication for gotu kola use is weight loss, and the type of liver injury on histopathology is granulomatous acute hepatitis with varying severity of necrosis and risk of progression to chronic liver disease.^[56]^ Gotu kola-induced acute liver failure in a young girl who took it (20 mg/day) for the treatment of acne was also reported. The authors stated that pentacyclic triterpene derivatives, which are naturally present in herbs, potentiate hepatotoxicity.^[57]^ (Fig. 5A).
2. Indian senna, *Cassia angustifolia* Vahl. belonging to the Fabaceae family *are* popularly used as over-the-counter laxatives and in traditional medical practice. Some researchers have also considered senna a safe herbal alternative for weight loss. The major bioactive components are anthraquinone glycosides and anthracoids (sennosides A and B). Animal studies in rodent models, as well as clinical investigations, have clearly revealed that senna toxicity is associated with enhanced hepatotoxicity serum markers along with the presence of necrotic lesions in the liver.^[58]^ Sennosides are converted into rhein anthrons by gut bacteria, which are further glucuronidated, sulfated, and excreted through feces and urine. Rhein anthron is structurally like danthron, a well-known hepatotoxic laxative. The anthraquinones in rhubarb, a major component of the rhein anthron, have been shown to be associated with liver injury.^[59]^ Senna use has been reported to be associated with severe hepatocellular and cholestatic liver injury and possible renal injury due to the presence of anthraquinone alkaloids. Liver histology usually reveals hepatocellular necrosis along with moderate-to-severe mixed cellular-type lobular inflammation.^[60–62]^ Metabolite profiling has shown that the hepatotoxicity of senna species is related to the sennoside B and A fractions.^[63]^ Acute liver failure due to consumption of senna-based herbal tea, portal vein thrombosis due to consumption of boiled, dried Indian senna leaves in a 42-year-old woman without underlying comorbid disease or prothrombotic conditions, and senna-related fatal acute liver failure in an elderly woman were reported in the medical literature.^[64–66]^ Senna in the form of dietary supplements or laxatives could result in severe hepatocellular liver injury with the potential for progression to fatal acute liver failure in predisposed patients (Fig. 5B).
3. *Garcinia cambogia* Desr. (Malabar tamarind), as per Ayurvedic medicine, is “claimed” to be a safe remedy for constipation, rheumatic diseases, helminthic infestation, and as a weight-loss agent due to its appetite-suppressing activity. Malabar tamarind is a fruit-bearing plant native to South and Southeast Asia. It is a fruit that is typically used in flavoring diets and as a food preservative. The bioactive metabolite hydroxycitric acid (HCA) reduced fatty acid synthesis, reduced lipogenesis, increased fat loss, and promoted appetite suppression in preclinical studies and isolated case series.^[67]^ Nonetheless, the medical literature is currently replete with liver toxicity associated with *Garcinia* either in the form of concentrated supplements or as part of multiherbal formulations, raising concerns.^[67,68]^ The liver injury is idiosyncratic, and the duration of use prior to the onset of symptoms ranged from 2 to 150 days, averaging around 7 to 28 days in most reports.^[69,70]^ The dose leading to liver injury varies, but fulminant liver failure has been notable with 2000 mg/day and a shorter duration of use.^[68,70]^ *Garcinia* supplements have been reported to cause acute hepatocellular or cholestatic hepatitis associated with varying patterns of necrosis, including confluent and sub-massive necrosis, and giant cell hepatitis resulting in subacute or acute liver failure, leading to death or necessitating liver transplantation^[70]^ (Fig. 5C).
4. *Morinda citrifolia* L. (Noni or Indian mulberry) is a perennial plant originating in Southeast Asia that contains more than 200 phytochemical substances with bioactive properties, including alcohols, phenols, anthraquinones, carotenoids, triterpenoids, flavonoids, glycosides, lactones, sterols, and aromatic compounds. Dietary supplements in the form of beverages (juice drinks), powders (from dried fruits), oil (from seeds), and leaf powders are available over the counter, promoting a wide range of therapeutic benefits exaggeratingly extrapolated from preclinical studies without conclusive evidence from clinical trials.^[71,72]^ Nonetheless, in rodent studies, Noni was shown to promote maternal hepatotoxicity, anti-implantation effects, intrauterine growth restriction, fetal abnormalities, deterioration of liver histology in the form of hepatocyte necrosis, reduction in liver volume, hepatic inflammation, disruption of synthetic activity (albumin reduction), and clinically relevant injury symptoms such as hypoactivity, excessive grooming, sunken eyes, and hunched posture, ultimately resulting in approximately 40% mortality within 3 months in exposed animals.^[73,74]^ Furthermore, prolonged Noni use in diabetic rats resulted in better glycemic control at the cost of kidney damage.^[75]^ From 2005 until now, 10 cases of non-induced liver injury have been reported worldwide, including severe hepatotoxicity in a 14-year-old boy.^[76–80]^ Liver pathology commonly demonstrates acute hepatitis with portal inflammation and periportal or other severe types of hepatocellular necrosis, hepatocellular cholestasis, ductular proliferation, and numerous eosinophils.^[79]^ In reported cases, after Noni berry-induced hepatotoxicity, 1 patient needed a liver transplant, while the majority of the others recovered on their own with supportive care. There is a wide time interval (4 weeks to 4 months) between the consumption of Noni berries and the onset of symptoms. The fruit portion, which is rich in anthraquinones, produces oxygen-derived free radicals, causes oxidative stress, and is hypothesized to be the cause of hepatotoxicity. Oxygen-derived free radicals deplete intracellular reduced glutathione and mitochondrial membrane potential, resulting in lipid peroxidation and, eventually, cell death^[76,79]^ (Fig. 5D).
5. Pyrrolizidine alkaloid-containing herbs include *Heliotropium* sp., *Trichodesma* sp., *Crotalaria* sp. (most used in Ayurveda), *Chelidonium majus* sp., *Holarrhena* sp., *and Castilleja* sp. Pyrrolizidine alkaloids (PAs) are amino acid-derived, nitrogen-bearing molecules derived from ornithine, and are commonly found as esters formed by a necine base (amino alcohols) and 1 or more necic acids (mono- or aliphatic dicarboxylic acids).^[81]^ Once ingested, dehydropyrrolizidine alkaloids (DHPAs) are important intermediary metabolites of pyrrolizidine alkaloids. DHPA binds to the sulfur, nitrogen, and oxygen groups in proteins to form adducts that enter the nucleus and react with DNA, resulting in genotoxicity and abnormal cellular function, primarily in the liver, leading to hepatocyte damage. These adducts pass to the adjacent space of Dissé as well as the sinusoidal lumen, where they injure the sinusoidal cells, leading to hepatic sinusoidal obstruction syndrome, a condition that can lead to acute or sub-acute liver failure potentiating organ transplantation or progression to cirrhosis and portal hypertension.^[82]^ DHPA can also reach the lung arterioles, causing secondary pulmonary hypertension and congestive heart failure in the long run.^[83]^ Tricodesmine, a byproduct of PA metabolism, is neurotoxic and can cause encephalitis, vertigo, stupor, and coma.^[82]^ The discovery that PA-containing herbs can accelerate the development of hepatic sinusoidal obstruction syndrome stems from reports of a large number of sporadic instances in Africa and the Indian subcontinent among persons consuming herbal teas and folk/traditional healer herbal remedies. Cross-contamination of herbal teas with PA-rich herbs, which results in liver damage, has also been described. Consumption of processed honey (during pollen drying) by *Crotalaria juncea* L. has been shown to induce PA-related hepatotoxicity in India.^[83]^ Similarly, consumption of *Holarrhena antidysenterica* (G.Don) Wall. ex A. DC. herb (known as “kutaja”) prescribed in Ayurvedic practice mostly for diabetes control as well as indigestion, piles, and worm infestation, leading to sinusoidal obstruction syndrome in a young male, and proof that rats fed with similar herbal extracts developed liver toxicity in the form of injury to centrilobular veins, centrilobular sinusoidal hemorrhage, congestion, and centrilobular and focal hepatocellular necrosis compatible with PA-induced damage have been reported.^[58,84]^ We recently transplanted a 56-year-old man with subacute hepatic failure secondary to sinusoidal obstruction due to folk-medicine use over 2 weeks, given for management of “anxiety.” (Fig. 6).

## 5. Herb-induced liver failure due to complementary and alternative medicine

**Figure 7.**
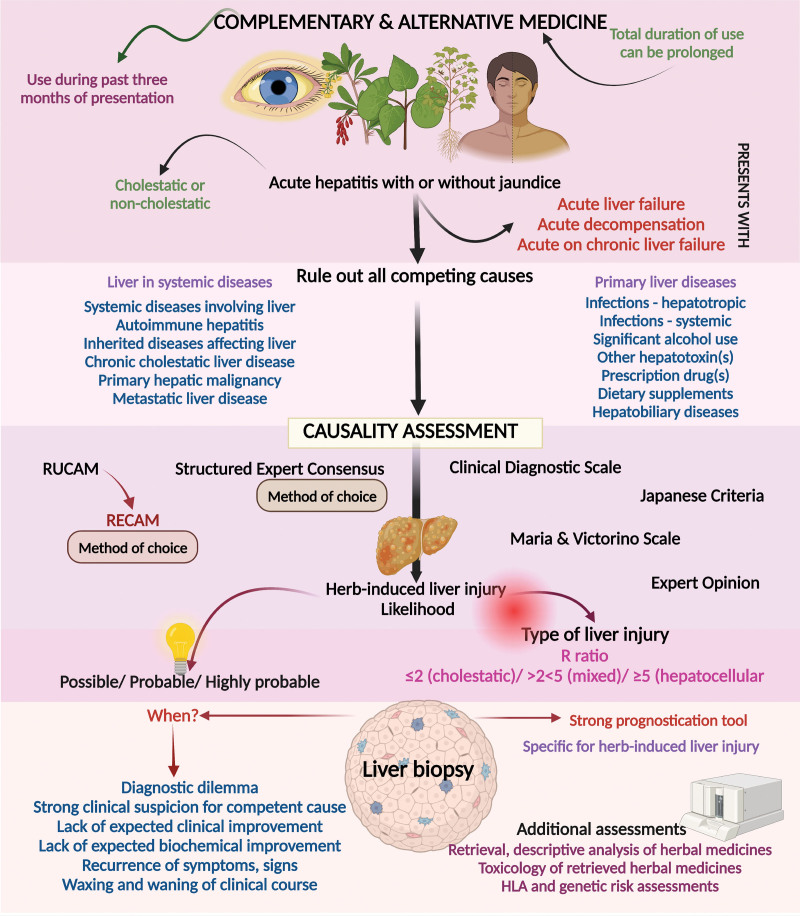
Algorithmic approach to a person with complementary and alternative medicine-related liver injury.

Acute liver failure (ALF) due to herbs, although commonly reported in the literature, has etiological agents that vary widely from region to region. In the United States, ALF is predominantly due to multiherbal formulations followed by herbal medicines containing black cohosh, horny goat weed, kava kava, and herbs belonging to traditional Chinese medicine (TCM), followed by herbal and dietary supplements (HDS). The need for liver transplantation was more frequent among patients with liver injury caused by herbal medicines compared to prescription drugs, with a lower 21-day transplant-free survival in the former.^[85]^ Similarly, the need for liver transplantation due to HILI triggered by HDS has increased greatly in the United States and among Asian populations utilizing TCM in the last decade.^[14]^ Across the published spectrum of HILI, the diagnosis has always been based on rational exclusions of competing causes (Fig. 7).

A pooled analysis of case reports revealed that approximately 4% and 9% of the patients died from liver failure and liver transplantation, developing HILI. The most implicated agents included proprietary branded HDS, *Polygonum multiflorum* Thunb. (Chinese climbing knotweed), green tea, and kava supplements.^[86]^ A large multicenter study revealed that ACLF due to drugs in the Asia-Pacific context was mostly due to CAM, particularly TCM.^[87]^ In the Indian context, ALF and ACLF have been well-described with the use of Giloy as well as Ashwagandha in multicenter studies.^[21,33]^ Nonetheless, Ayurvedic multiherbal formulations remain the most common cause from a CAM perspective that triggers ALF and ACLF in the Indian subcontinent. The death rate was close to 20% in patients who developed ALF due to Ayurvedic herbals in a single-center series. Hepatic encephalopathy at admission, presence of low albumin level, severe liver necrosis on liver histopathology, and presence of high levels of arsenic and mercury in ingested products were independent predictors of mortality due to ALF.^[7]^ Similarly, HILI due to Ayurvedic medications resulted in ACLF in more than one-third of the patients with cirrhosis who used CAM, of whom 53% died in the absence of timely liver transplantation. The most used formulations were multiherbal drugs, many of which were unlabeled or traditional herbs containing A. vera, guava leaf, passion flower leaf, bitter oleander, and metallo-mineral preparations. Baseline hepatic encephalopathy, total bilirubin, hyponatremia, leukocytosis, and grade and severity score of ACLF predicted death at follow-up up to 12 months.^[9]^ Further prospective studies including larger patient cohorts from different regions in India to clearly define the causative agents and predictors of clinical outcomes in ALF or ACLF due to herbs and herbal supplements remain unmet.

## 6. Summary and conclusion

We have exhaustively reviewed the common but globally impactful herbs utilized in the Indian Ayush system of medicine with the potential for hepatotoxicity to guide physicians in making a thorough clinical history review of patients who present with acute onset liver disease and related complications in the context of drug/HILI. The narrative that CAMs, especially those from the Indian subcontinent, negatively impact health due to adulteration and contamination, has changed over the last decade.^[88]^ Even though contamination and adulteration of alternative medicines, especially Ayurvedic formulations, remains a major public health concern, we must also realize the potential for direct toxicity, especially on the liver, of traditionally used natural components in single herb or multiherbal formulations.^[58]^ Heavy metal contamination of Ayurvedic herbals and adverse impacts on human health, such as cluster poisoning due to lead, are well known from large case–control and prospective cohort studies, including qualitative studies from India.^[89,90]^ Even though the need to analyze an herbal formulation that has caused liver injury for contamination and adulteration remains a knee-jerk reaction, it is imperative to look for potential direct hepatotoxic herb(s) in the formulation rather than investing in a resource-squandering chemical/toxicology analysis. The current published literature is richer in knowledge regarding the potential toxicities of various herbs commonly used in traditional medicine practices in India as well as other regions across the globe.^[91]^ Turmeric, Ashwagandha, and Giloy are herbs with a high risk of hepatobiliary toxicity that are consistently reported in the medical literature. Furthermore, beyond the scope of this review, various other herbal and dietary supplements that are part of both traditional and nontraditional over-the-counter use have been reported to have severe hepatotoxic potential.^[86]^ Efforts must be made to educate the public as well as practitioners of CAM to remain vigilant of avoidable disease burdens within the community by practicing critical thinking towards evidence-based health-seeking behavior and following rationale and logic driven by empirical evidence in the context of preventive and therapeutic management, respectively.

## Author contributions

**Conceptualization:** Cyriac Abby Philips.

**Visualization:** Cyriac Abby Philips.

**Writing—original draft:** Cyriac Abby Philips.

**Writing—review & editing:** Cyriac Abby Philips, Arif Hussain Theruvath.

**Validation:** Arif Hussain Theruvath.

## Supplementary Material








