# Supplementary material for: A comprehensive review on the hepatotoxicity of herbs used in the Indian (Ayush) systems of alternative medicine
Source: Medicine (Baltimore). 2024 Apr 19;103(16):e37903. doi: 10.1097/MD.0000000000037903 (PMC11029936; doi:10.1097/MD.0000000000037903)
Supplement: Supplementary file 1 [file medi-103-e37903-s001.docx]

**Supplementary table 1:** Pertinent studies on *Tinospora cordifolia* (Giloy)-related liver injury

| **No.** | **Author/ Year/ Patients** | **Liver injury and associated features** | **Clinical outcomes and comments** |
| --- | --- | --- | --- |
| 1 | Nagral A et al./ 2021/ N=6 | Liver injury was predominantly in women, four patients had underlying chronic liver disease  Median duration of consumption of herb as fresh plant or proprietary tablet formulation was 90 days  Various types of autoantibodies related to liver disease strongly positive  Hepatocellular and cholestatic mixed pattern with autoimmune hepatitis-like features with lymphoplasmacytic inflammation along with eosinophils, varying grades of interface hepatitis and necrosis | One patient with acute-on-chronic liver failure died  Majority were on long-term immunosuppression therapy  Giloy herb unmasked autoimmune hepatitis in those with silent disease and triggered autoimmune hepatitis in those at risk for autoimmune liver disease |
| 2 | Gupta S et al./ 2022/ N=2 | One male and one female  One consumed fresh plant and the other in form of pellets available over-the-counter from market  Various types of autoantibodies related to liver disease strongly positive  Hepatocellular and cholestatic mixed pattern with bridging or sub-massive necrosis | One patient developed acute liver failure and underwent liver transplantation  Catastrophic liver failure associated with short term use of Giloy-herb in persons without additional risk factors |
| 3 | Parikh P/ 2022/ N=2 | Both females  Consumed capsules of Giloy-herb extracts available in market  Various types of autoantibodies related to liver disease strongly positive  Liver injury was associated with lymphocytic inflammation with necrosis and severe interface hepatitis | Short course of immunosuppression therapy led to complete resolution of acute hepatitis  Giloy-herb can trigger de-novo autoimmune hepatitis which requires finite treatment with immunosuppressive agents |
| 4 | Gupta H et al./ 2022/ N=2 | Both women  Various types of autoantibodies related to liver disease strongly positive  Consumed fresh plant boiled water decoction and commercially available Giloy-herb tablets for one month  Biopsy showed expanded portal tracts and interface hepatitis with moderate mixed inflammatory infiltrates comprising neutrophils, eosinophils, plasma cells, and lymphocytes and varying severity of liver cell necrosis | Both patients improved with short course of steroids without any relapse of autoimmune liver disease on follow up  The importance of herb-induced autoimmune hepatitis compared to flare of primary autoimmune hepatitis is emphasized |
| 5 | Sahney et al./ 2022/ N=3 | Authors discuss three severe cases out of suspected 25 cases due to Giloy herb-induced liver injury  All three were women in whom two had underlying autoimmune disorders (hypothyroidism and systemic lupus)  Liver injury was hepatocellular or mixed and associated lymphoplasmacytic infiltration, numerous eosinophils and in portal tracts and interface hepatitis | All survived, one patient was on long term immunosuppression due underlying chronic liver disease.  One needed only withdrawal of offending herb that resulted in spontaneous resolution of hepatitis |
| 6 | Gupta D et al./2022/N=3 | Two females and one male  All had hepatocellular pattern of liver injury  Intake of branded tablets of giloy herb extract or home-made boiled herb decoctions  No liver biopsy was performed and all patients were managed with symptomatic care only | All patients survived with resolution of hepatitis only on withdrawal of offending herb  Corticosteroids were not used, but hepatitis resolution and recovery time were prolonged  The immunomodulatory properties of Giloy herb can lead to autoimmune-like illness in predisposed persons and trigger autoimmune liver disease in at-risk groups |
| 7 | Rastogi M et al./ 2022/ N=2 | Two patients without any underlying comorbidities or known chronic illness  Giloy used for 2-4 weeks duration leading to severe jaundice and hepatitis  Biopsy did not show features of autoimmune liver injury and revealed perivenular necrosis and mild portal infiltrate, no interface hepatitis, paucity of plasma cells, no rosettes, and relatively mild-moderate inflammatory infiltrate, predominantly with eosinophils | Both patients responded to short course of steroids and ursodeoxycholic acid therapy  One patient self-re-challenged with the Giloy formulation and developed new onset and fluctuating jaundice which resolved spontaneously a second time also, but after a prolonged disease course |
| 8 | Kulkarni A et al./2022/N=43 | More than half of cohort were females  Median time from initial Giloy consumption to symptom onset was 46 days  Patients presented with acute hepatitis, acute worsening of chronic liver disease (most common clinical presentation), or acute liver failure  Liver biopsy revealed acute hepatitis with autoimmune features and hepatocyte and canalicular cholestasis and neutrophilic and eosinophilic infiltration | The largest published series of cases of Giloy‐induced acute hepatitis  Approximately 10% patients died within two months of presentation, and nearly 5% required liver transplantation within 90 days  Analysis of retrieved Giloy samples did not reveal any competing hepatotoxic agents  Positive autoimmune markers in acute (non-viral) hepatitis may indicate triggered or unmasked autoimmune hepatitis for which active reasons must be sought, and should include recent or long‐term exposure to herbals containing Giloy |
| 9 | Nnamani I et al./ 2023/ N=1 | 50-year-old female with autoimmune thyroiditis  Consumed HistaEze^TM^ supplement containing *Tinospora cordifolia*  No liver biopsy was done | Short course of steroids significantly reduced hepatitis  Liver injury resolved completely in 90 days |
| 10 | May K et al./ 2023/ N=1 | 54-year-old woman without any comorbid illnesses  Consumed Giloy powder along with other herbal drugs for three months for gastrointestinal complaints  No liver biopsy was performed | Withdrawal of offending herbals resolved hepatitis spontaneously  No steroids given and only symptomatic care provided |
