# Supplementary material for: A comprehensive review on the hepatotoxicity of herbs used in the Indian (Ayush) systems of alternative medicine
Source: Medicine (Baltimore). 2024 Apr 19;103(16):e37903. doi: 10.1097/MD.0000000000037903 (PMC11029936; doi:10.1097/MD.0000000000037903)
Supplement: Supplementary file 2 [file medi-103-e37903-s002.docx]

**Supplementary table 2:** Pertinent studies on *Withania somnifera* (Ashwagandha)-related liver injury

| **No.** | **Author/ Year/ Patients** | **Liver injury and associated features** | **Clinical outcomes and comments** |
| --- | --- | --- | --- |
| 1 | Björnsson HK et al./ 2020/ N=5 | Liver injury was predominantly in men, mean age 43 years  Median duration of herb intake and symptom onset was 2-12 weeks  Pruritus and jaundice were most common presentations  Pain abdomen is a distinct feature  Liver histology classically reveals cholestatic hepatitis | All patients survived and hepatitis resolved within 1-5 months  None developed chronic liver injury  Recovery from cholestatic hepatitis was prolonged in most, but without further clinical consequences |
| 2 | Ireland PJ et al./ 2021/ N=1 | 39-year-old female  154mg of Ashwagandha root extract on alternate days for six weeks for anxiety  No cholestatic symptoms at onset but later developed severe abdominal pain and itching  Liver biopsy revealed features of a severe acute cholestatic hepatitis with confluent necrosis but no convincing signs of chronicity | Started on ursodeoxycholic acid and symptoms abated in two weeks’ time  But complete resolution of jaundice was after a prolonged course  No recurrence of symptoms |
| 3 | Weber S et al./ 2021/ N=1 | Male  450mg ashwagandha daily for one year and 20 days  Severe cholestatic hepatitis  No liver biopsy was performed | Complete resolution on withdrawing herbal supplement  No recurrence or chronicity  Authors also discuss close to 100 cases of self-reported adverse events related to ashwagandha use from product websites main featuring pruritus and abdominal pain symptoms |
| 4 | Sajjadh MJ et al./ 2022/ N=1 | 20-year-old male  Jaundice, pruritus, and pain abdomen  OTC Ashwagandha 450mg once daily for 30 days  No liver biopsy was performed | Spontaneous resolution on withdrawal of offending herbal supplement  OTC medication and herbal supplements containing Ashwagandha are unregulated and caution must be exercised |
| 5 | Rattu M et al./ 2022/ N=1 | 44-year-old female  Daily Ashwagandha supplement for ‘anxiety’  Severe cholestatic jaundice with abdominal pain  No liver biopsy was performed | Symptomatic care with cholestyramine improved liver tests and hepatitis resolved in few weeks’ time  No re-challenge was performed |
| 6 | Lubarska M et al./ 2023/ N=1 | Male gender  Consuming only ashwagandha for one year  Progressive cholestatic jaundice, hospitalization after 90 days of symptom onset  No liver biopsy was performed | Severe cholestatic symptoms and progressive and stormy course of jaundice  Required therapeutic plasma exchange and intravenous steroids to improve liver tests and reduce symptoms  Three months after treatment, hepatitis resolved |
| 7 | Bokan G et al./ 2023/ N=2 | Both women  In the first case, ashwagandha capsules (450 mg, three times daily) for six months; second case, 45 days of using ashwagandha capsules (450 mg)  Severe cholestatic hepatitis without chronicity  Elastography of liver showed no fibrosis | Improvement in liver enzyme levels two weeks after the cessation of ashwagandha capsules and complete normalization after four and eight weeks. |
| 8 | Vazirani S et al./ 2023/N=1 | 48-year-old man  Severe cholestatic liver injury – ashwagandha along with alcohol use  Predominant symptom was pain abdomen followed by jaundice and pruritus  Liver biopsy was not performed | Progressive improvement in symptoms and liver tests ensued after two weeks  Hepatitis resolved after one month on only supportive care  No recurrence of symptoms even though patient relapsed on alcohol after one month |
| 9 | Suryawanshi et al./ 2023/ N=1 | 41-year-old woman  Prescribed unknown dose of ashwagandha and progesterone by a Naturopath for general well-being and improvement in quality of life after thyroid resection  Severe progressive hepatocellular jaundice leading to acute liver failure, no cholestatic symptoms | First report to demonstrate acute liver failure due to ashwagandha supplements  Patient survived after receiving a liver transplantation  Extensive hepatocellular necrosis with acute hepatitis on liver histology |
| 10 | Tóth M et al./ 2023/N=1 | 65‐year‐old woman  Began to take ashwagandha, because of “troubled thoughts” four weeks before admission  Liver biopsy revealed perivenular spotty hepatocellular necrosis, accompanied with multiple ceroid‐laden macrophages, hepatocellular, and canalicular cholestasis | Following withdrawal of the drug, the serum transaminase activities and serum bilirubin gradually dropped without any further specific therapy  Only a symptomatic therapy with cholestyramine was administered on the second day due to pruritus  Three months later, all liver tests were normal |
| 11 | Philips CA et al./ 2023/ N=8 | Only patients with ashwagandha single-ingredient formulation-related herb-induced liver injury included  Male predominant  Cholestatic hepatitis was the commonest presentation  Liver biopsy revealed cholestatic features predominantly with hepatocellular necrosis and lymphocyte/eosinophil predominant portal-based inflammation  Chemical analysis of retrieved formulations revealed only natural phytochemicals without adulteration or contamination | Largest series of ashwagandha-related liver injury in published literature  Five patients had underlying chronic liver disease; three presented with acute-on-chronic liver failure, and all three died on follow-up  One patient progressed to chronic herb-induced liver injury  Ashwagandha-liver injury presents with cholestatic hepatitis and can lead to the syndrome of acute-on-chronic liver failure with high mortality in those with pre-existing liver disease |
