# Supplementary material for: A comprehensive review on the hepatotoxicity of herbs used in the Indian (Ayush) systems of alternative medicine
Source: Medicine (Baltimore). 2024 Apr 19;103(16):e37903. doi: 10.1097/MD.0000000000037903 (PMC11029936; doi:10.1097/MD.0000000000037903)
Supplement: Supplementary file 3 [file medi-103-e37903-s003.docx]

**Supplementary table 3:** Pertinent studies on *Curcuma longa* [Turmeric (or its bioactive compound curcumin)]-related liver injury

| **No.** | **Author/ Year/ Patients** | **Liver injury and associated features** | **Clinical outcomes and comments** |
| --- | --- | --- | --- |
| 1 | Lukefahr et al./ 2018/ N=1 | Elderly woman  Underlying metabolic syndrome, Raynaud’s syndrome, irritable bowel syndrome  Turmeric supplement intake for eight months  Autoantibody (p-ANCA) was positive  Biopsy showed neutrophils, lymphocytes, and plasma cells-based moderate to marked portal inflammation with focal piecemeal portal necrosis  Subsequent reassessment of the liver biopsy by the authors revealed auto fluorescent inclusions in the pigment-laden histiocytes with an excitation/emission spectrum consistent with curcumin, a turmeric-derived polyphenol or possibly lipofuscin | Abnormal liver tests significantly decreased within 30 days of discontinuation of turmeric supplement, which normalised by 13 months and have remained normal even after three years follow-up |
| 2 | Luber RP et al./ 2019/ N=2 | One male and one female  One patient had re-challenge and liver injury recurred  None had significant comorbidities  Liver biopsy (done in one patient) showed florid inflammatory cell infiltrate comprising lymphocytes, histiocytes, neutrophils, scattered eosinophils, and minimal plasma cells in the portal tracts with interface hepatitis  The turmeric supplement tested negative for drugs, adulterants, or toxic heavy metals | Both patients survived  Liver injury resolved after withdrawal of offending herbal supplement  Complete resolution of hepatitis in one month  No recurrence of hepatitis was noted on follow up |
| 3 | Imam Z et al./ 2019/ N=1 | Elderly female  No significant comorbidities  One month use of curcumin-only supplement (500mg)  Hepatocellular type of liver injury  No liver biopsy was performed | Complete resolution of hepatitis in 42 days  No re-challenge was performed  No recurrence of hepatitis on follow-up |
| 4 | Gasbarrini G et al./ 2019/ N=4 | 4 patients, aged 25–45 years, 3 females and 1 male  Hypertransaminasemia up to 3–6 times with respect to normal value, and increasing of gamma glutamyl transpeptidase up to 4–8 times the norm was found | Only one of 4 cases had coagulation failure  Hepatocyte necrosis and cholestasis regressed after the spice withdrawal  Only in one case hepatitis lasted for 20 days |
| 5 | Abdallah MA et al./ 2020/ N=1 | 51-year-old woman  Daily use 500 mg of turmeric DS capsules (each capsule containing: 400 mg of turmeric powder, 50 mg of turmeric extract, and 50 mg of organic ginger powder) for 45 days  Hepatitis associated with myalgias and fatigue  Liver biopsy demonstrated moderate mixed  inflammation consisting of neutrophils, eosinophils,  and lymphocytes involving the portal areas | Abnormal liver tests reduced by 50% in six days and complete resolution of hepatitis in eight weeks  No recurrence of hepatitis noted during follow-up period |
| 6 | Chand S et al./ 2020/ N=1 | 62-year-old woman  Treated breast cancer and in remission  Turmeric tablets for 10 months for “wellness”  Hepatitis was associated with myalgias, fatigue and a widespread urticarial rash on her limbs and torso.  Biopsy demonstrated inflammation of the parenchyma with neutrophils, mononuclear cells, plasma cells and occasional eosinophils, with focal parenchymal necrosis and mild cholestasis | Following cessation of the turmeric supplement (on presentation), her liver function tests improved dramatically within a fortnight  Complete resolution in less than six weeks |
| 7 | Suhail et al./2020/ N=1 | 61-year-old female  Underlying polycystic liver disease  Turmeric supplements for six months  Hepatitis associated with polyarthralgia  Hepatocellular type of liver injury  Autoimmune antibody (ANA) was positive  Biopsy showed pan lobular hepatitis with early parenchymal collapse suggestive of acute hepatitis and hepatocellular pattern of injury along with ceroid laden macrophages | Turmeric supplements were discontinued and the hepatitis resolved in 21 days with steroid treatment  No recurrence of hepatitis during follow-up period |
| 8 | Lee BS et al./ 2020/ N=1 | 55-year-old woman  Underlying Hashimoto’s thyroiditis, autoantibody (ANA) strongly positive  Qunol Liquid Turmeric 15 mL daily for 90 days  Liver biopsy showed interface hepatitis with a mixture of plasma cells, lymphocytes, eosinophils, and neutrophils and features were consistent with autoimmune hepatitis | Withdrawal of turmeric supplement drastically improved liver tests and complete resolution of hepatitis in one month without recurrence of hepatitis  No immunosuppression therapy started  Case highlights triggered autoimmune hepatitis due to turmeric (herb-induced autoimmune hepatitis) |
| 9 | Sohal A et al./ 2021/ N=2 | Both women – first, over-the-counter supplement containing turmeric 2000 mg and black pepper daily for three months for back pain, second, turmeric tablets two times a day, duration unknown  Hepatocellular type of liver injury  Liver biopsy in first revealed hepatocyte ballooning and necrosis, with an absence of periportal necrosis, steatosis, or fibrosis and in the second, done two months after the initial presentation, nonspecific patchy, predominantly mononuclear portal inflammation without readily identifiable interface activity and clusters of ceroid-laden macrophages  Autoantibody ANA was positive in the second patient | Complete resolution in six months in the first patient and within one month in the second patient |
| 10 | Lombardi N et al./ 2021/ N=7 | Records of Tuscan cases of acute hepatitis were obtained from the Italian Phytovigilance system  In all cases, hepatotoxicity was associated with Curcuma longa formulations with high bioavailability and high dosage of curcumin/curcuminoids  Predominantly in females (6/7), time to onset 1-8 months, positive de-challenge successful in 5/7 patients  Doses ranged from 400mg to 1000mg of whole turmeric extracts, 1-4 tablets a day | In the 23 cases identified through the systematic review, most patients were concomitantly exposed to at least one other medication  None of the patients died and all improved with withdrawal of offending herbal supplement and supportive care |
| 11 | Prisca P et al./ 2022/ N=1 | 62-year-old female with a history of hypertension  Consumption of turmeric tea over the preceding three weeks  Hepatocellular type of liver injury  Liver biopsy was not performed and the dose of turmeric tea consumption was not delineated | The patient improved with turmeric cessation, with complete resolution of abnormal liver enzymes on follow-up |
| 11 | Sunagawa SW et al./ 2022/ N=1 | 49-year-old woman with a history of ocular hypertension, polycystic ovarian syndrome  Two capsules of turmeric supplement (1000mg containing 950mg curcuminoids and 10mg black pepper) for 1.5 months  Hepatocellular type of liver injury  A liver biopsy was not performed | Follow-up visit 71 days after the initial presentation demonstrated complete resolution of her jaundice and liver chemistries  No immunosuppressive therapies were initiated at any point during her treatment. |
| 12 | Ajitkumar A et al./ 2023/ N=1 | 55-year-old woman  Alcohol use, two glasses of wine daily for 30 years, last intake five days prior to jaundice onset  Turmeric supplements 1500 mg once daily for wrist pain for one month  No liver biopsy was performed | Spice withdrawal along with intravenous n-acetyl cysteine infusion resolved symptoms and ameliorated liver test abnormalities  Her liver function tests normalized two months after her initial presentation |
| 13 | Smith DN et al./ 2023/ N=1 | 62-year-old female with hypertension  Drank turmeric tea for three weeks prior to symptom onset (dosage not identified)  No liver biopsy was performed | The patient improved clinically with turmeric cessation, and her liver enzymes returned to normal range upon follow-up after one month. |
| 14 | Halegoua-DeMarzio D et al./ 2023/ N=10 | Eight were women and median age was 56 years  Liver injury was hepatocellular type in nine patients and mixed in one  Liver biopsies in four patients showed acute hepatitis or mixed cholestatic-hepatic injury with eosinophils  Chemical analysis confirmed the presence of turmeric in all seven products tested; three also contained piperine (black pepper) | Five patients were hospitalized, and one patient died of acute liver failure  HLA typing demonstrated HLA-B*35:01 as risk factor  Turmeric causes potentially severe liver injury that is typically hepatocellular, with a latency of 1 to 4 months and strong linkage to HLA-B*35:01 |
