# Supplementary material for: A comprehensive review on the hepatotoxicity of herbs used in the Indian (Ayush) systems of alternative medicine
Source: Medicine (Baltimore). 2024 Apr 19;103(16):e37903. doi: 10.1097/MD.0000000000037903 (PMC11029936; doi:10.1097/MD.0000000000037903)
Supplement: Supplementary file 4 [file medi-103-e37903-s004.docx]

**Supplementary table 4:** Pertinent studies on *Psoralea corylifolia* (Bakuchi/ Babchi)-related liver injury

| **No.** | **Author/ Year/ Patients** | **Liver injury and associated features** | **Clinical outcomes and comments** |
| --- | --- | --- | --- |
| 1 | Chinese Pharmacopoeia Committee – 1978 to 2005/ N=11 | Total 11 cases of liver injury associated with the use  of proprietary Chinese medicines for the treatment of vitiligo in the Chinese literature with psoralen, isopsoralen, and coumestrol being the main constituents – typically seen in Bakuchi  The typical liver injury was a hepatocellular injury pattern with onset after approximately three weeks | There are no data on therapeutic or toxic dosage of psoralen in human |
| 2 | Nam SW et al./ 2005/ N=1 | 44-year-old female  Several packs of Bakuchi along with a cup of black tea daily for seven weeks (over 10 times the usual dosage)  No pre-existing liver disease, no autoantibodies were positive  Severe acute cholestatic hepatitis  Liver biopsy showed confluent necrosis of hepatocytes in zone 3 region with haemorrhage, infiltration of inflammatory cells, and bile stasis | The patient was discharged with improved condition at day 10  Complete normalization of liver tests 133 days after the development of initial symptoms |
| 3 | Cheung WI et al./ 2009/ N=3 | 39- and 22-year-old men and 20-year-old woman  Patient 1: Proprietary herbal products “Xin Cu Hei Su Sheng Zhang Ji” and “Qu Bai Ba Bu Qi Pian” for two months for vitiligo - s three to five tablets thrice daily  Patient 2: Two herbal products for vitiligo namely, “Bu-gu-zhi intramuscular injection,” 2 mL alternate day, and “Qu Bai Ba Bu Qi Pian,” four tablets thrice daily for two months. (The main ingredient in Bu-gu-zhi injection preparation is Bakuchi)  Patient 3: Herbalist prescribed powdered herbs for two weeks in the form of powder consisting of Bakuchi 1.3 g along with other traditional Chinese herbs  Non-cholestatic jaundice  Liver biopsy was not performed | All three patients had spontaneous resolution of symptoms and acute hepatitis after discontinuation of the herbal supplement and supportive care with 4-8 weeks’ time |
| 4 | Teschke R et al./ 2009/N=1 | 64-year-old female  Self-treatment with various Ayurvedic herbals including Bakuchi for nine months for vitiligo bought from a clinic in India through online consultation  Severe cholestatic hepatitis  Liver biopsy was not performed | Complete normalization of liver tests and symptom resolution in 130 days with only discontinuation of herbs and supportive care |
| 4 | Smith DA et al./ 2014/ N=1 | 52-year-old woman  Bakuchi seeds direct intake for an unknown period and an unknown dose for vitiligo  The liver biopsy showed a cholestatic acute hepatitis | Complete resolution of hepatitis after withdrawal of offending herb |
| 5 | Aujla AK et al./ 2019/ N=1 | 60-year-old woman  Bakuchi seeds four weeks prior to presentation and would apply it topically and consume it daily  Positive anti-smooth muscle antibodies  Developed acute liver failure  Biopsy revealed submassive necrosis and collapse; hepatitis with plasma cells and eosinophils with significant cholestasis. | Acute liver failure improved spontaneously and so did not require liver transplantation  Was started on steroids for short term and tapered and stopped  Complete resolution of hepatitis after one month |
| 6 | Li A et al./ 2019/ N=1 | 53-year-old woman  Consumed Qubaibabuqi tablets for seven months for vitiligo  Acute cholestatic hepatitis | Rapid progression to acute liver failure, multiple organ failure and death within five days of symptoms onset  First reported case of fatality from Bakuchi herbal treatment |
| 7 | Gandhi HJ et al./ 2023/ N=1 | 44-years-old woman  Rheumatoid arthritis and vitiligo  Consuming Bakuchi seeds (twice a day) for six months  Acute cholestatic jaundice with ascites  Liver biopsy showed marked intrahepatic cholestasis with ballooning and feathery degeneration, focal interface activity, broad fibrous septa with fibrosis, marked ductular reaction, dense lymphoplasmacytic inflammatory infiltrate admixed with eosinophils, and neutrophils with areas of collapse of liver parenchyma suggestive of herb-induced cholestatic hepatitis in the background of cirrhosis | First case of acute-on-chronic liver failure related to Bakuchi use  Was listed for liver transplantation, but eventually recovered from progressive liver failure  Was given a short course of immunosuppression with steroids and low-dose azathioprine  Complete recompensation after one month follow-up  Herb-induced liver injury secondary to Bakuchi seeds even though rare, can cause worsening of underlying liver disease |
